# Organisation of the endosperm and endosperm–placenta syncytia in bladderworts (*Utricularia*, Lentibulariaceae) with emphasis on the microtubule arrangement

**DOI:** 10.1007/s00709-012-0468-5

**Published:** 2012-11-20

**Authors:** Bartosz J. Płachno, Piotr Świątek, Hanna Sas-Nowosielska, Małgorzata Kozieradzka-Kiszkurno

**Affiliations:** 1 Department of Plant Cytology and Embryology, Jagiellonian University, 52 Grodzka St., 31-044 Cracow, Poland; 2 Department of Animal Histology and Embryology, University of Silesia, 9 Bankowa St., 40-007 Katowice, Poland; 3 Department of Plant Anatomy and Cytology, University of Silesia, 28 Jagiellońska St., 40-032 Katowice, Poland; 4 Department of Plant Cytology and Embryology, University of Gdańsk, 59 Wita Stwosza St., 80-308 Gdańsk, Poland

**Keywords:** Plant reproduction, Embryology, Cellular endosperm, Haustorium, *Utricularia*, Lentibulariaceae, Aquatic carnivorous plants

## Abstract

Multinucleate cells play an important role in higher plants, especially during reproduction; however, the configurations of their cytoskeletons, which are formed as a result of mitosis without cytokinesis, have mainly been studied in coenocytes. Previous authors have proposed that in spite of their developmental origin (cell fusion or mitosis without cytokinesis), in multinucleate plant cells, radiating microtubules determine the regular spacing of individual nuclei. However, with the exception of specific syncytia induced by parasitic nematodes, there is no information about the microtubular cytoskeleton in plant heterokaryotic syncytia, i.e. when the nuclei of fused cells come from different cell pools. In this paper, we describe the arrangement of microtubules in the endosperm and special endosperm–placenta syncytia in two *Utricularia* species. These syncytia arise from different progenitor cells, i.e. cells of the maternal sporophytic nutritive tissue and the micropylar endosperm haustorium (both maternal and paternal genetic material). The development of the endosperm in the two species studied was very similar. We describe microtubule configurations in the three functional endosperm domains: the micropylar syncytium, the endosperm proper and the chalazal haustorium. In contrast to plant syncytia that are induced by parasitic nematodes, the syncytia of *Utricularia* had an extensive microtubular network. Within each syncytium, two giant nuclei, coming from endosperm cells, were surrounded by a three-dimensional cage of microtubules, which formed a huge cytoplasmic domain. At the periphery of the syncytium, where new protoplasts of the nutritive cells join the syncytium, the microtubules formed a network which surrounded small nuclei from nutritive tissue cells and were also distributed through the cytoplasm. Thus, in the *Utricularia* syncytium, there were different sized cytoplasmic domains, whose architecture depended on the source and size of the nuclei. The endosperm proper was isolated from maternal (ovule) tissues by a cuticle layer, so the syncytium and chalazal haustorium were the only way for nutrients to be transported from the maternal tissue towards the developing embryo.

## Introduction

Fuso-morphogenesis, the formation of multinucleated syncytia by cell–cell fusion, plays an important role in tissue development and organogenesis in animals and plants. It acts, e.g. during the development of animal muscles (myotubes), bones (multinucleate osteoclasts) and placentae (syncytiotrophoblast). In addition, it also occurs during embryo and postembryonic organ formation, for example in the pharynx, hypodermis, vulva and uterus in the model invertebrate species *Caenorhabditis elegans* (see Shemer and Podbilewicz 2000, 2003; Podbilewicz 2005 and literature cited therein). Multinucleate cells also play an important role in higher plants, especially during reproduction; the most common, however, are coenocytes, which are formed as a result of mitosis without cytokinesis (young stage of a gymnosperm and angiosperm female gametophyte, the nuclear endosperm, some endosperm haustoria and commonly the anther tapetum, non-articulated laticifers) (e.g. Johri 1984; Serpe et al. 2001; Reiser and Fischer 1993; Brown et al. 1994, 2002; Baluška et al. 2004a, b; Brown and Lemmon 2008). In the literature, they are frequently referred to as syncytial structures, such as a syncytial endosperm (e.g. Otegui and Staehelin 2000; Nguyen et al. 2002). In this paper, as in our recent paper (Płachno and Świątek 2011), the term syncytium is strictly limited to identifying the multinucleate structure derived from cells that have fused together (definition sensu Baluška et al. 2004a), and these are not gametes. The main examples of such structures, which occur during angiosperm development, are the articulated laticifers, the amoeboid tapetum, the nucellar plasmodium in the species of the Podostemaceae family and the special heterokaryotic placenta–endosperm syncytia of *Utricularia* (see Płachno and Świątek 2011 and literature cited therein).

It has been proposed that in spite of their developmental origin (cell fusion or mitosis without cytokinesis), in multinucleate plant cells, radiating microtubules determine the regular spacing of individual nuclei—‘cell bodies’. Thus, each individual nucleus in these multinucleate cells controls its own cytoplasmic domain. It has been suggested that the size of these domains depends on the DNA content and nuclear volume (Brown and Lemmon 1992, 2001; Baluška et al. 2004a, b, 2006). However, to date, the evidence supporting this thesis has come mainly from objects with homokaryotic coenocytes with nuclei of a similar size and origin, e.g. a nuclear type of endosperm (Brown et al. 1999; Otegui and Staehelin 2000; Brown and Lemmon 2007) or from a *Ginkgo* female gametophyte (Brown et al. 2002). The only heterokaryotic syncytia that have been studied were the heterokaryotic syncytia induced by parasitic nematodes. The depolymerisation of microtubules takes place in those syncytia, which may decrease the viscosity of the cytoplasm and helps nematodes in nutrient absorption (de Almeida Engler et al. 2004). Data about the architecture of the microtubule cytoskeleton in heterokaryotic plant syncytia with nuclei of different sizes and origins that are different from ‘natural’ plant heterokaryotic syncytia are still missing.

The syncytia formed in *Utricularia* arise from different progenitor cells: cells of the maternal sporophytic nutritive tissue and the micropylar endosperm haustorium (both maternal and paternal genetic material). These syncytia contain two classes of nuclei—two giant nuclei from the endosperm haustorium and several small nuclei from nutritive tissue cells. Recently, the ultrastructure and development of these syncytia were analysed in *Utricularia intermedia* (Płachno and Świątek 2011). This study showed that the *Utricularia* endosperm–placenta syncytium can be considered to be a giant transfer cell.

The main aim of this study was to characterise the organisation of endosperm and endosperm–placenta syncytia of two *Utricularia* species (*Utricularia minor* and *U*. *intermedia*) with emphasis on the microtubule arrangement in these structures. Because the formation of *Utricularia* syncytia using transmission electron microscopy has only been studied in *U*. *intermedia* (Płachno and Świątek 2011), there is a need to follow this process in other species. In this paper, we also describe the formation and ultrastructure of the syncytia of *U*. *minor*. Recently, the actin cytoskeleton has been analysed in the syncytia of *Utricularia* (Płachno et al. 2011), so we were able to compare the configuration of both cytoskeletal domains in the studied tissues.

## Materials and methods

### Plant material

Flowers and fruits of *U*. *minor* L. and *U*. *intermedia* Hayne (sect. *Utricularia*) were obtained from the Jeleniak-Mikuliny Nature Reserve near the town of Lubliniec (Płachno and Świątek 2008). Additional fruits of *U*. *minor* were collected near Třeboň (Třeboň Biosphere Reserve), Czech Republic.

### Electron microscopy studies

For the electron microscopy studies, placentae with ovules and young seeds were isolated from ovaries and fixed in 2.5 % formaldehyde and 2.5 % glutaraldehyde in a 0.05-M cacodylate buffer (pH 7.0) for 2 days. The material was postfixed in 1 % OsO_4_ in a cacodylate buffer for 24 h at ~4 °C, rinsed in the same buffer, treated with 1 % uranyl acetate in distilled water for 1 h, dehydrated with acetone and embedded in an Epoxy Embedding Medium Kit (Fluka). Semithin sections were stained with methylene blue and examined using an Olympus BX60 microscope. Ultrathin sections were cut on a Leica ultracut UCT ultramicrotome. After contrasting with uranyl acetate and lead citrate, the sections were examined using a Hitachi H500 electron microscope at 75 kV.

### Visualisation of nuclei and microtubules

For the visualisation of nuclei and microtubules, placentae with ovules and seeds were fixed in a mixture of 4 % formaldehyde (freshly prepared from paraformaldehyde) and 0.25 % glutaraldehyde in a piperazine buffer overnight at 4 °C. After fixation and three washes in a buffer, they were dehydrated in a graded MeOH series. The material was then infiltrated with Steedman’s wax (Vitha et al. 2000). Then, 5–10-μm sections were taken from the embedded ovaries and adhered to poly-l-lysine-coated microscope slides. The sections were dried overnight, dewaxed in ethanol, rehydrated in an ethanol–phosphate-buffered saline (PBS) series and rinsed in PBS. Tissue sections were preincubated in 1 % bovine serum albumin in PBS for 45 min, washed in PBS and then incubated in a monoclonal antibody against α tubulin (Rat mAb [YOL1/34] ab6161 (Abcam) 1:800 solution) overnight at 4 °C. The sections were then washed in PBS and incubated for 4 h in a secondary goat pAb to rat IgG [FITC] ab6840-1 (Abcam) 1:600 solution antibody. The slides were rinsed in PBS and the nuclei were stained with 4′,6-diamidino-2-phenylindole dihydrochloride (7 μg/1 ml, Sigma). The slides were then mounted in an anti-fading solution (CITIFLUOR glycerol/PBS solution AF1 R1320 Christine Gropl or in the mixture of PBS/glycerol 1:1). In the control experiments, which were conducted in a similar manner but omitting the first antibody, no tubulin staining was detected. Fluorescence was examined using an Olympus BX60 epifluorescence microscope. The chosen slides were examined using an Olympus FV1000 confocal system equipped with an Olympus IX81 inverted microscope. More than 200 ovules and seeds with tissue of both species were examined; in many cases, we detected a strong autofluorescence of cytoplasm in nutritive tissue cells and syncytia, which impeded a good visualisation of the cytoskeleton. In many syncytia, we failed to visualise microtubules at all, despite the fact that in the tissues surrounding the syncytia and ovule tissues strong immunoreaction signals were observed. Quantitative fluorescence measurements on acquired datasets could support our presented results. Performing such measurements was considered by the authors when establishing experimental procedures; however, methodological constrictions made it impossible to perform them. Any quantitative analysis of fluorescence intensity on microscopical samples requires maintaining invariable conditions of fluorescence emission and data acquisition. To fulfil this demand, ideally, the samples should be collected, fixed, stained and illuminated in a single experimental procedure. In case of our material fulfilling, this requirement is nearly impossible because of problems in obtaining, proper fixing and staining the plants. On the other hand, comparing the fluorescence data acquired in different time points requires data normalisation. This means that the internal fluorescence standard should be used—a fluorochrome at a known concentration, which has very good light efficiency and stably emits fluorescence. To the authors’ knowledge, there is no such commercially available standard and there are no protocols for plant material suggesting what could be used as an internal standard.

The quality of fixation was checked using the differential interference contrast microscopy technique. It should be underlined that our studied species were not an ideal material for study. They belong to affixed aquatic *Utricularia* species, which are difficult in cultivation in laboratory conditions; thus, flowers and fruits have to be collected from the natural environment. Both *U*. *intermedia* and *U*. *minor* are protected by Polish law and also by Czech Republic law and were collected from the protected areas: Jeleniak-Mikuliny Nature Reserve and Třeboň Biosphere Reserve. So in the future, other aquatic *Utricularia* species which are easier in cultivation should be used for the syncytium studies.

## Results

**Fig. 1:**
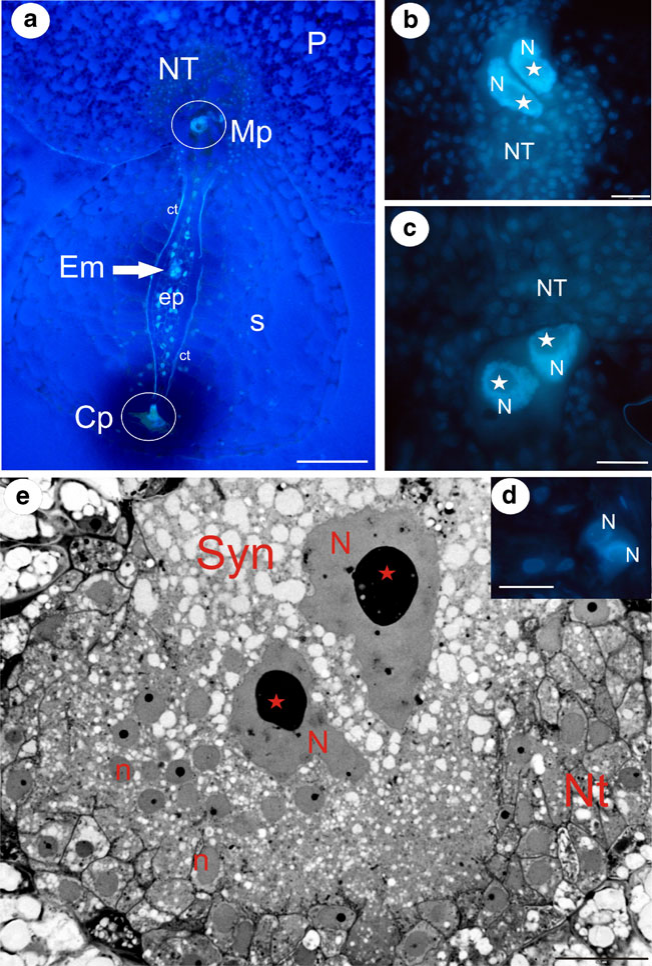
Development of the endosperm and syncytium in *Utricularia*: **a** Longitudinal section through the placenta and seed of *Utricularia intermedia* showing the relationship between the different endosperm domains and the proembryo; nutritive tissue (*NT*), placenta (*P*), micropylar endosperm part (*Mp*), endosperm proper (*ep*), proembryo (*Em*), cuticle (*ct*), seed (*s*), chalazal endosperm part (*Cp*), *bar* = 100 μm. **b**, **c** The micropylar endosperm haustoria of *Utricularia minor*; nutritive tissue (*NT*), nucleolus (*star*), *bar* = 20 μm. **d** The chalazal endosperm haustorium of *Utricularia minor*; nutritive tissue (*NT*), nucleolus (*star*), *bar* = 20 μm. **e** Section through the *Utricularia minor* syncytium; syncytium (*Syn*), giant endosperm nucleus (*N*), nucleolus (*star*), nucleus from the nutritive tissue cell (*n*), nutritive tissue (*Nt*), *bar* = 20 μm

The development of the endosperm in the species studied is very similar; therefore, the following descriptions refer to both species unless indicated otherwise. In the *Utricularia* species analysed, the endosperm development after fertilisation was according to the Scutellaria type. Early on, when a proembryo had only a few cells, the endosperm was composed of three domains: the micropylar (Mp), central (endosperm proper) and the chalazal (Cp) (Fig. 1a). Only the endosperm proper was separated from the ovule tissue by a clearly visible cuticle layer of the integumental tapetum (Fig. 1a). The micropylar domain was formed by a large haustorium, which was connected with nutritive placental cells. As a rule, the apical part of the haustorium was occupied by two nuclei, each having one large nucleolus (Fig. 1b, c). The chalazal part of the endosperm also formed a haustorium, which always had two nuclei. In contrast to the micropylar haustorium nuclei, those nuclei were much smaller (Fig. 1d).

**Fig. 2:**
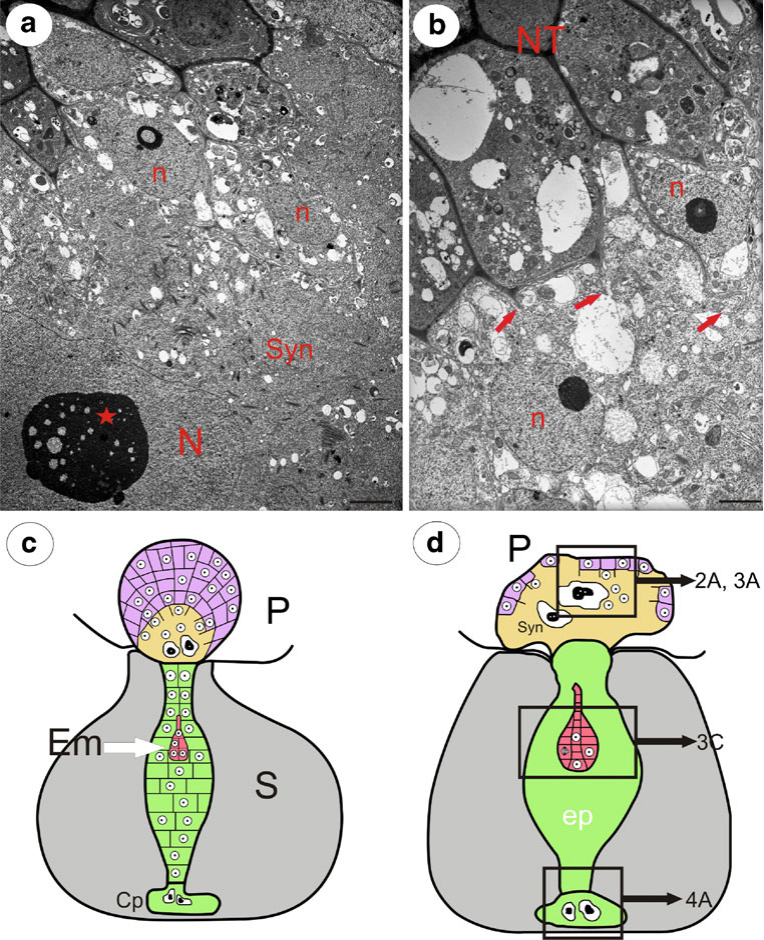
Sections through the mature syncytium of *Utricularia minor* and schematic drawings of the young *Utricularia* seeds. **a** Micrograph showing the ultrastructure of the syncytium with two different populations of nuclei; syncytium (*Syn*), giant endosperm nucleus (*N*), nucleolus (*star*), nucleus from nutritive tissue cell (*n*), *bar* = 2 μm. **b** The peripheral part of the syncytium, where the protoplasts of nutritive cells merge with the syncytium; nutritive tissue (*NT*), *arrows*—digested nutritive tissue cell walls, nucleus from nutritive tissue cell (*n*), *bar* = 1.2 μm. **c**, **d** Schematic drawings of two developmental stages of young *Utricularia* seeds, showing relationships between the endosperm–placenta syncytium, chalazal haustorium, endosperm proper and embryo; placenta (*P*), seed (*S*, *gray color*), nutritive tissue (*violet color*), endosperm proper (*ep*, *green color*), embryo (*Em*, *red color*), syncytium (*Syn*, *yellow color*), Cp (chalazal endosperm haustorium). **d** In the endosperm proper, the individual cells were not shown. Note that in the older stage (**d**), only small amount of the nutritive tissue persists, and the syncytium is fully formed and occupied the place of the nutritive tissue. Framed parts mark corresponding photographs shown in **a** in this figure and in Figs. 3a, c and 4a

**Fig. 3:**
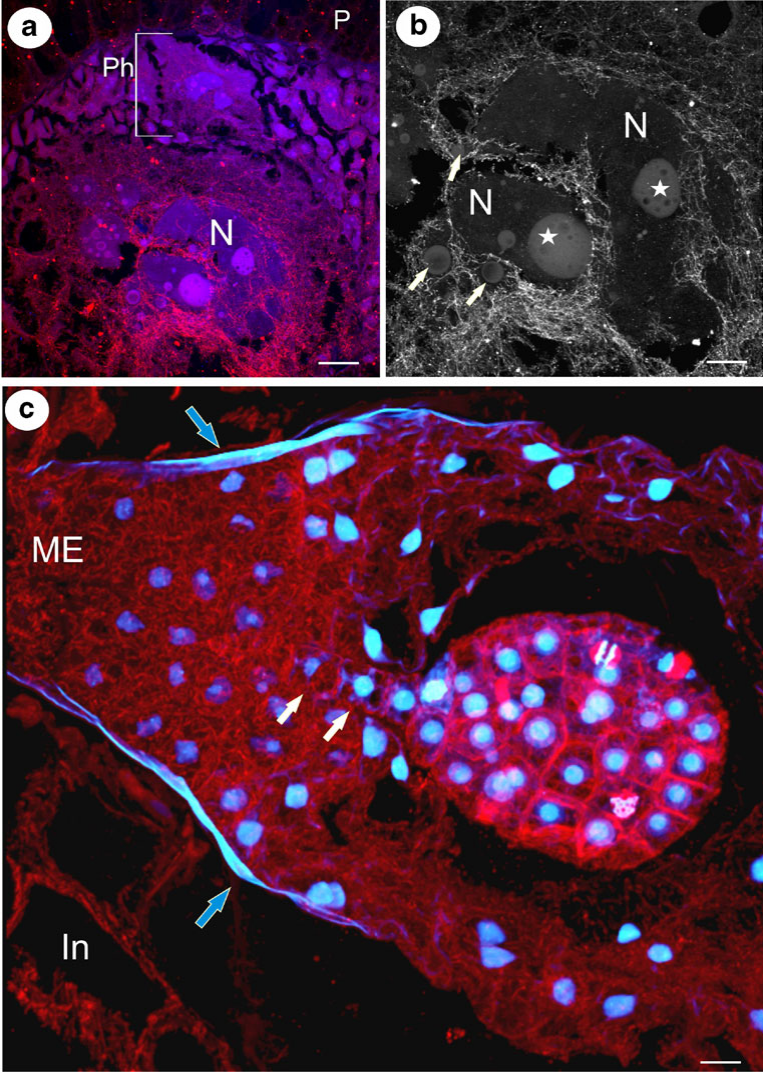
Visualisation of microtubules in the syncytium, endosperm and embryo. **a** Arrangement of the microtubule cytoskeleton in the syncytium of *Utricularia intermedia*; the peripheral part of the syncytium with nuclei from the nutritive tissue cells (*Ph*), two giant endosperm nuclei in the central part of the syncytium (*N*), placenta (*P*), *bar* = 36 μm. **b** The microtubular cage between the lobes of two giant endosperm nuclei syncytium of *Utricularia intermedia*; giant endosperm nucleus (*N*), nucleolus (*star*), additional nucleoli (*arrows*), *bar* = 18 μm. **c** The architecture of the microtubule cytoskeleton in the endosperm proper and embryo of *Utricularia minor*; micropylar part of endosperm proper (*ME*), cuticle (*arrows*), suspensor’s cells (*white arrows*), integument (*In*), *bar* = 10 μm

As the development of the embryos progressed, the endosperm also expanded; there were mitotic divisions in the endosperm proper, and both haustoria increased in size. Finally, the cell walls separating the micropylar haustorium from the nutrient tissue cells were digested and a heterokaryotic syncytium formed (Fig. 1e). Within the fully formed syncytium, two giant nuclei originated from the micropylar haustorium, while the rest were from the nutritive tissue cells (Fig. 2a–d). In *U*. *minor*, similar to *U*. *intermedia*, the united cytoplasm of the mature syncytium was stratified into two main zones: a central zone where two giant nuclei of an endosperm origin occurred and a marginal zone where new protoplasts of the nutritive cells joined the syncytium (Figs. 1e and 2a). In the mature syncytium of *U*. *minor*, the giant nuclei from the endosperm haustorium were lobed in contrast to *U*. *intermedia*, where the giant nuclei were more or less amoeboid in shape (Fig. 3a).

**Fig. 4:**
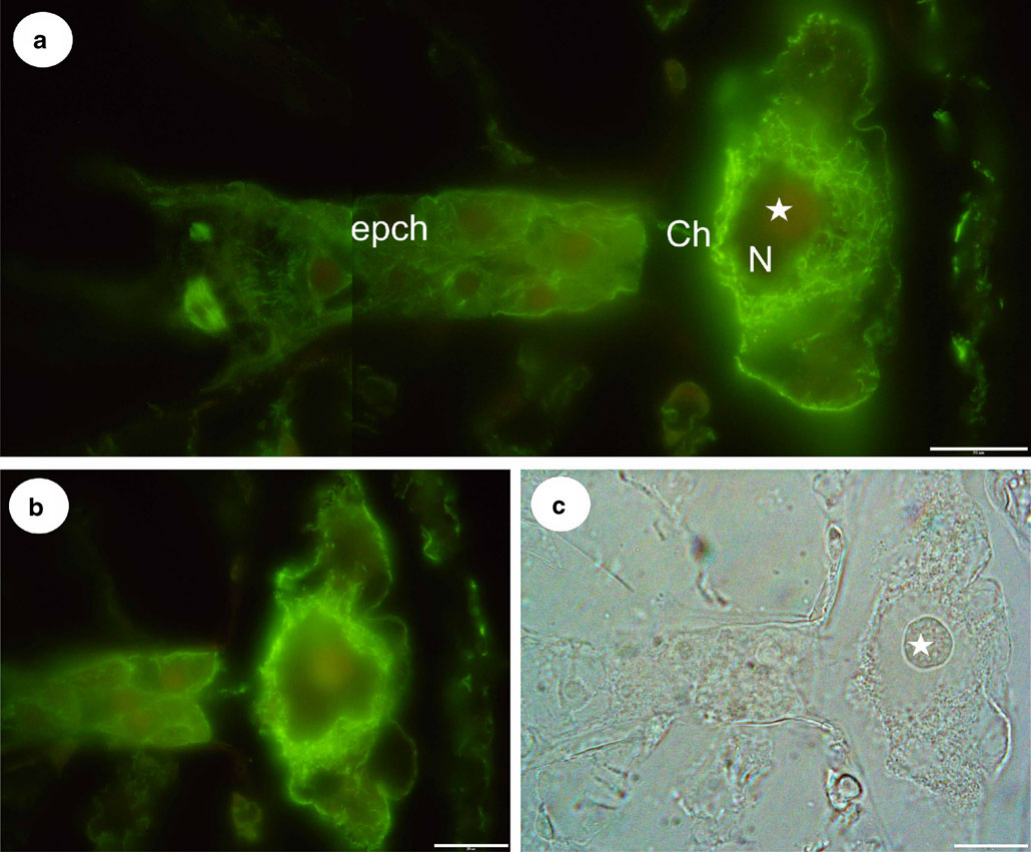
The microtubule arrangements and structure of the chalazal endosperm haustorium of *Utricularia minor*. **a**, **b** Visualisation of the microtubules in the chalazal part of the endosperm proper and the haustorium; the chalazal part endosperm proper (*epch*), haustorium (*Ch*), haustorium nuclei (*N*), nucleolus (*star*), *bar* = 20 μm. **c** Visualisation of the chalazal endosperm haustorium using differential interference contrast; nucleolus (*star*), *bar* = 20 μm

In the endosperm–placenta syncytia, both giant nuclei were surrounded by a three-dimensional cage created by the microtubules as was demonstrated by anti-tubulin staining (Fig. 3a). Microtubules occurred close to the nuclear envelope, and additionally, numerous bundles of microtubules penetrated the spaces between the lobes of the giant nuclei (Fig. 3b). These two pools of microtubules were intermingling. Each giant nucleus had one huge nucleolus with numerous small vacuoles in the central part (Fig. 2a). Within the lobes of the giant nuclei in *U*. *intermedia*, numerous additional smaller nucleoli were found (Fig. 3b). At the marginal zone where new protoplasts of the nutritive cells joined the syncytium, microtubules formed a network which surrounded the nuclei of the former nutritive cells and which were also distributed through the cytoplasm (Fig. 3a). Several microtubule bundles were seen in the cytoplasmic bridges between the vacuoles in the syncytia (not shown). Microtubules were organised into a special dense meshwork of bundles at the chalazal part of the syncytium adjacent to the wall separating the syncytium from the micropylar endosperm cells (Fig. 3a). The endosperm micropylar proper cells were highly cytoplasmic (a dense cytoplasm with numerous organelles) with abundant microtubules within the cells (Fig. 3c). These cells surrounded the suspensor of the embryo. In embryo cells, an extensive network of cortical cytoskeleton (during interphase), microtubular spindles during mitosis and phragmoplast during cytokinesis was observed (Fig. 3c). The globular embryo possessed a long filamentous suspensor (Fig. 3c). Within the suspensor cells, the microtubules formed a well-developed cortical network (Fig. 3c). The embryo proper was surrounded by endosperm cells which had a poorly developed cortical microtubule network. In contrast, the chalazal endosperm cells had a rich microtubular cytoskeleton, which formed a prominent network through the cytoplasm (Fig. 4a). In the chalazal endosperm haustorium, a huge concentration of the microtubule bundles was visible near the nuclei, but some cortical microtubules were also visible (Fig. 4a–c).

To reiterate, five major types of the microtubular cytoskeleton organisation were observed in the material studied:

1. A dense meshwork of microtubules concentrated around the nucleus–giant nuclei cell bodies.
2. A rather loose microtubular cytoskeleton with microtubules present in the cytoplasm and around the nucleus–protoplasts of the newly joined nutritive cells.
3. A very rich, dense microtubular cytoskeleton with microtubules evenly distributed in the cytoplasm and around the nucleus—endosperm micropylar proper cells.
4. A microtubular cytoskeleton organised mainly in the form of the cortical cytoskeleton—i.e. embryo proper.
5. A dense microtubular meshwork around the nuclei of the chalazal endosperm haustorium; cortical microtubules also occur.

The type of microtubule cytoskeleton organisation was tissue and cell type specific.

## Discussion

The endosperm has been the model tissue in studies devoted to mitotic spindle organisation in plant cells. Especially, the study of the African blood lily (*Haemanthus katherinae*) endosperm expanded knowledge about the mitotic spindle, phragmoplast and cell plate formation (e.g. Bajer 1965, 1968; Bajer and Allen 1966; Forer and Jackson 1979; De Mey et al. 1982).

In flowering plants, three major types of endosperm development, nuclear, ab initio cellular and helobial, have been recognised (Vijayaraghavan and Prabhakar 1984). The most important agriculture species (cereals, Fabaceae), ornamental *H*. *katherinae* and also the model plant *Arabidopsis thaliana*, which have the nuclear endosperm type and cytoskeleton configuration during endosperm development of these species, have been described in detail (e.g. De Mey et al. 1982; Molè-Bajer and Bajer 1983; Bajer and Molè-Bajer 1986a, b; Webb and Gunning 1991; Brown et al. 1994, 2003; Brown and Lemmon 2007; XuHan and van Lammeren 1994; Otegui and Staehelin 2000; Olsen 2001). To date, immunoreaction investigations on the microtubular cytoskeleton in other endosperm types have been done in the orchid *Cymbidium sinense* (Huang et al. 1998), but in this case, the endosperm development appeared to be stagnant and had no influence on later embryo development. To the best of our knowledge, our study is the first published paper about the microtubular cytoskeleton configurations in the ab initio cellular endosperm. We showed the diversity of microtubule configurations in the three functional endosperm domains: the micropylar syncytium, the endosperm proper and the chalazal haustorium. Such a functional endosperm specialisation, in the case of the cytoskeleton, was observed in the species from the Brassicaceae family with a nuclear endosperm type (Nguyen et al. 2001, 2002; Brown et al. 2003). Thus, functionally, the chalazal *Utricularia* haustorium can be compared with the chalazal large multinucleate cyst in the genera *Lepidium*, *Coronopus* and *Arabidopsis*, which plays an important role in loading maternal resources (nutrients) into the developing seed (Nguyen et al. 2000, 2001). This could also be the case in *Utricularia* seeds, in which the nutritional materials are mainly stored in the embryo cell, and not in the endosperm (Płachno and Świątek 2010). The development of the endosperm proper as well as embryogenesis in *Utricularia* represents a typical vegetative growth when considering (with respect to) cytoskeleton configurations (Webb and Gunning 1991; Figs. 1–5 in Brown and Lemmon 2001). We found that the *Utricularia* endosperm micropylar proper cells, with their dense cytoplasm and abundant microtubules, were a transition region between the syncytium and the central part of the endosperm proper. According to Farooq (1964), the cells of this endosperm part may have a haustorium character and even fuse with the syncytium.

### Nuclear cytoplasmic domains

It is generally acknowledged that the size of the nucleus and the cell cytoplasmic volume are interconnected; however, the nature of this interaction remains unclear. Two main concepts have been proposed—one suggesting that the nuclear volume and/or its DNA content determine the volume of the cytoplasm, and another which proposes that the cytoplasm (mainly the abundance of the plasma membranes and proteins) is responsible for limiting the nuclear volume (Webster et al. 2009). There are arguments for and against both theories, though they come mainly from studies on animal cells. In plants, most of the data come from studies of syncytia from the species that develop homokaryotic syncytia, i.e. the cereal’s nucellar endosperm (Brown et al. 1994; Brown and Lemmon 2007; XuHan and van Lammeren 1994), the central part of the *Arabidopsis* nucellar endosperm (Brown et al. 2003) and the *Ginkgo* female gametophyte (Brown et al. 2002). In these species, the coenocytes are organised into regular, nuclear cytoplasmic domains (cell bodies—sensu Baluška et al. 2004a, b), which are similar in size. Unfortunately, data from heterokaryotic coenocytes are missing. Our research on *Utricularia* provides evidence supporting the hypothesis that the size of the nuclear cytoplasmic domains/cell bodies depends on the volume of nuclei (Baluška et al. 2004a, 2006). However, we did not observe the radial systems of microtubules around nuclei in the syncytia, but a complicated meshwork of microtubules concentrated around these nuclei. We show that there are different sized cytoplasmic domains in the *Utricularia* syncytium, whose architecture depends on the source and size of the nuclei. The syncytium studied is formed by cell fusion coupled with cell wall lysis. Our data show that the cytoplasmic bodies formed by those newly integrated into the syncytium cells do not change their cytoplasmic volume, which is delimited by the microtubular cytoskeleton, after joining the syncytium (thus, when the cells lose their cell walls, their cytoplasm becomes a part of the syncytium). This suggests that the size of the nucleus may be important for the determination of cytoplasmic volume. However, cytoplasmic factors cannot be excluded (Webster et al. 2009).

### The microtubular cytoskeleton in giant plant polyploid cells

Apart from the nuclear endosperm mentioned, there is a little information concerning the microtubular cytoskeleton of the giant polyploid cells in flowering plants. To date, investigations on these types of cells have only been performed on the huge haustorial suspensor in *Sedum* (Kozieradzka-Kiszkurno et al. 2011), the suspensors in orchids (Ye et al. 1997; Huang et al. 1998), as well as the giant cells and heterokaryotic syncytia that are induced by parasitic nematodes (de Almeida Engler et al. 2004). It was suggested that in suspensor cells, a well-developed microtubular cytoskeleton may play an important role during their expansion and elongation (Ye et al. 1997; Huang et al. 1998; Kozieradzka-Kiszkurno et al. 2011).

Interestingly, in *Sedum acre*, the microtubules congregate near the huge nucleus of the basal suspensor cell. This microtubule configuration also occurs in both the chalazal endosperm haustorium and the endosperm–placenta syncytia of *Utricularia*. This may be explained by the fact that in plant cells, the nuclear envelope is responsible for microtubule nucleation. It is also worth noting that recently in *Arabidopsis*, AtSUN and AtWIP have been identified, which are the counterparts of the animal SUN-domain and KASH-domain proteins, respectively (Boruc et al. 2012; Oda and Fukuda 2011). In animals, the SUN-KASH protein bridges are responsible for connecting the microtubular cytoskeleton with nucleoskeleton (lamins) and have been shown to be involved in the determination of the nuclear shape, nuclear location or chromatin regulation and most probably also play the same role in plants (Boruc et al. 2012; Graumann and Evans 2010). Moreover, the data supporting the presence of lamina-like proteins in plants are increasing (Dittmer et al. 2007; Fiserova et al. 2009; van Zanten et al. 2011). Thus, it is tempting to speculate that the three-dimensional microtubular cage observed in *Utricularia*, which surrounds the nucleus, may be responsible for the nuclear positioning and/or the determination of its shape, as was proposed for some specialised animal cells and nurse cells in insect ovaries (Biliński and Jaglarz 1999; Żelazowska and Biliński 2001 and literature cited therein). On the other hand, data from studies of the *Allium* cell nucleus suggest an actin-dependent rotation and translocation of the nucleus within the cell (Chytilova et al. 2000). Experiments with cytoskeleton destabilising drugs show that actin filaments but not microtubules are involved in nucleus migration and positioning in the filamentous plant cells (Lloyd et al. 1987; Ketelaar et al. 2002). However, studies from animals show that the nuclear envelope has the protein complexes that are responsible for interacting with both types of cytoskeletal elements; thus, both microtubules and actin filaments may play a role in nuclear regulation, though their roles may be different. In support of this, actin disruption did not affect either the nuclear shape or organisation of the nuclear grooves (Collings et al. 2000). Moreover, in contrast to an actin cytoskeleton, some cell types use microtubule-generated forces to actively maintain the shape of their nuclei (Webster et al. 2009). In the syncytia induced by parasitic nematodes, the microtubular cytoskeleton was strongly disturbed; however, in giant cells that were also induced by parasitic nematodes, there was mitotic division but with arrested phragmoplasts and without pre-prophase bands (de Almeida Engler et al. 2004).

In the endosperm–placenta syncytia of *U*. *intermedia*, two patterns of F-actin were observed (Płachno et al. 2011)—an extensive F-actin network around the giant nuclei and embedded in the syncytium nuclei of former nutritive cells, which was similar to the microtubular cytoskeleton configuration found in the present study. Additionally, actin bundles, which can support cytoplasmic streaming and perform routes for organelle motility, were found.

### The cuticle barrier between the embryo sac and maternal tissue

A cuticle layer between the embryo sac/endosperm and the adjacent integumental tapetum was recorded in many unrelated species (Ingram 2010), e.g. in the ovules of *Jasione montana* (Berger and Erdelská 1973), *Arabidopsis* (Beeckman et al. 2000) and in young seeds of *Trifolium repens* (Jakobsen et al. 1994). The thick layer of cutin-like material between the endosperm and integument was also observed in the members of the Crassulaceae family (Kozieradzka-Kiszkurno and Płachno 2012). Also in cereals, a cuticular material occurs between the endosperm and the testa tissue (Zee and O’Brien 1970). Similar to *Utricularia*, there was a lack of cuticle layer on the micropylar and chalazal poles in the examples mentioned. According to several authors (Jakobsen et al. 1994; Kapil and Tiwari 1978; Bouman 1984; Beeckman et al. 2000), the cuticle blocks the supply of nutrients to the embryo sac/endosperm. However, we think that in *Utricularia*, this cuticle layer may also play another function. We suggest that it protects maternal tissues from aggressive endosperm behaviour. On the micropylar and chalazal poles where the cuticle layer was absent, endosperm haustoria developed and destroyed maternal tissue; thus, the transfer routes for nutrients may occur in these places. Especially, those endosperm–placenta syncytia of *Utricularia* are characterised by hypertrophy of nuclei, abundant endoplasmic reticulum and organelles and the occurrence of wall ingrowths (Płachno and Świątek 2011). Thus, all of these characteristics indicate high activity and may support the nutritive and transport functions of the endosperm–placenta syncytium; however, more physiological or biochemical evidence is needed to draw a final conclusion.

## Conclusions

- The formation and ultrastructure of the *U*. *minor* syncytia is similar to *U*. *intermedia*.
- There were different sized cytoplasmic domains, whose architecture depended on the source and size of the nuclei, in the heterokaryotic endosperm placenta.
- Our results support the suggestion that the size of nuclear cytoplasmic domains (cell bodies) depends on the volume of nuclei.
- The endosperm proper was isolated from maternal tissues by a cuticle layer; therefore, the syncytium and chalazal haustorium played important roles in the loading of maternal resources (nutrients) into the developing embryo.
